# Reprogramming of blood cells into induced pluripotent stem cells as a new cell source for cartilage repair

**DOI:** 10.1186/s13287-016-0290-7

**Published:** 2016-02-17

**Authors:** Yueying Li, Tie Liu, Nicholas Van Halm-Lutterodt, JiaYu Chen, Qingjun Su, Yong Hai

**Affiliations:** Key Laboratory of Genomic and Precision Medicine, Beijing Institute of Genomics, Chinese Academy of Sciences, Beijing, 100101 China; Department of Orthopedics, Beijing Chao-Yang Hospital, Capital Medical University, GongTiNanLu 8#, Chaoyang District, Beijing, 100020 China; Clinical and Translational Research Center of Shanghai First Maternity & Infant Hospital, School of Life Sciences and Technology, Tongji University, Shanghai, China

**Keywords:** Peripheral blood cells, Induced pluripotent stem cells, Chondrocytes

## Abstract

**Background:**

An attempt was made to reprogram peripheral blood cells into human induced pluripotent stem cell (hiPSCs) as a new cell source for cartilage repair.

**Methods:**

We generated chondrogenic lineage from human peripheral blood via hiPSCs using an integration-free method. Peripheral blood cells were either obtained from a human blood bank or freshly collected from volunteers. After transforming peripheral blood cells into iPSCs, the newly derived iPSCs were further characterized through karyotype analysis, pluripotency gene expression and cell differentiation ability. iPSCs were differentiated through multiple steps, including embryoid body formation, hiPSC-mesenchymal stem cell (MSC)-like cell expansion, and chondrogenic induction for 21 days. Chondrocyte phenotype was then assessed by morphological, histological and biochemical analysis, as well as the chondrogenic expression.

**Results:**

hiPSCs derived from peripheral blood cells were successfully generated, and were characterized by fluorescent immunostaining of pluripotent markers and teratoma formation in vivo. Flow cytometric analysis showed that MSC markers *CD73* and *CD105* were present in monolayer cultured hiPSC–MSC-like cells. Both alcian blue and toluidine blue staining of hiPSC–MSC-chondrogenic pellets showed as positive. Immunohistochemistry of collagen II and X staining of the pellets were also positive. The sulfated glycosaminoglycan content was significantly increased, and the expression levels of the chondrogenic markers *COL2*, *COL10, COL9* and *AGGRECAN* were significantly higher in chondrogenic pellets than in undifferentiated cells. These results indicated that peripheral blood cells could be a potential source for differentiation into chondrogenic lineage in vitro via generation of mesenchymal progenitor cells.

**Conclusions:**

This study supports the potential applications of utilizing peripheral blood cells in generating seed cells for cartilage regenerative medicine in a patient-specific and cost-effective approach.

**Electronic supplementary material:**

The online version of this article (doi:10.1186/s13287-016-0290-7) contains supplementary material, which is available to authorized users.

## Background

Articular cartilage has a very poor intrinsic capacity for self repair, which poses a problem for regenerative medicine. Cartilage tissue engineering is a promising and potential directive to provide biological replacement tissue. This may require an adequate amount of seed cells and, as it is generally known, there is a limited source of naturally occurring autologous chondrocytes. Seed cells usually obtained from an ectopic cartilage source often require additional surgery which seems to be a significant challenge in clinical practice [1]. Adult stem cells appear to be a promising alternative cell source for cartilage tissue engineering; nevertheless, it is undoubtedly fraught with limitations. The percentage of mesenchymal stem cells (MSCs) in the bone marrow of an adult is rather low, and the yield of stem cells from older patients has also been reported to show relatively lower proliferative rates in culture. Some comorbidities may also affect the proliferative abilities of stem cells. Previous studies have postulated that MSCs from patients with osteoarthritis potentially show reduced chondrogenic differentiation [2].

Recently, human induced pluripotent stem cells (hiPSCs) generated from somatic cells established an innovative direction for cartilage repair. This basically introduces OCT3/4 and SOX2 along with either KLF4, c-MYC, NANOG, or LIN28 through viruses [3, 4]. Similar to embryonic stem cells, hiPSCs have pluripotency, proliferation capacity and gene expression without known ethical dilemma limitations. Moreover, new methods for generating iPSCs without viral vectors have also been developed in order to mitigate tumorigenicity [5, 6]. In addition to cell-based therapeutic applications, iPSC technology could also provide patient-specific cells and tissue models for tissue repair [7]. This may help overcome limitations associated with current cell harvest sources.

Many kinds of cells have been used to generate iPSCs. However, the most common somatic cells that have been extensively studied are fibroblasts which have been deeply explored for their possible reprogramming potentials; nevertheless, they possess some limiting properties. One of the limiting properties of primary fibroblasts includes the need for expansion into several passages in vitro*,* making it a questionable source for generating patient-specific stem cells [8]. An easy tendency for mutation may hinder the progress of application and further studies. Recently, umbilical cord blood and peripheral blood cells (PBCs) were found to be advantageous harvest sources for reprogramming. PBCs are commonly utilized in clinical applications and are scientifically and abundantly stored. PBCs are considered to be an ideal source, which might redirect focus of the research away from the skin to the blood. Zhang discovered efficient ways to generate iPSCs from human PBCs which makes it more accessible [9]. Recently, umbilical cord blood cells have been reported to be induced for differentiation into cardiac cells (cardiomyocytes) and hepatocytes [10, 11]. However, there is relatively scarce information on PBC reprogramming and differentiation into chondrocytes.

In this current study, we utilized PBCs as a source by reprogramming these cells to differentiate into chondrogenic lineage with a combination of a pellet culture system to mimic chondrocyte formation.

## Methods

### Blood sample and ethics statement

The use of human peripheral blood was approved by the Institutional Review Board (IRB). The IRB or Ethics Committee of Beijing Chao-Yang Hospital approved this study. The participants provided their written informed consent to participate in the study. The ethics committees or IRBs of Beijing Chao-Yang Hospital approved this consent procedure. This study was performed in accordance with the guidelines of the Animal Care and Use Committee of the National Institute of Biological Sciences and with the Guide for the Care and Use of Laboratory Animals. PBCs were either obtained from a blood bank or were freshly collected from volunteers. Peripheral blood mononuclear cells (PBMNCs; lymphocytes and monocytes) were obtained by density gradient centrifugation with Ficoll-paque plus (GE) at room temperature. The age of the healthy donors (male or female) ranged from 20–40 years. The human mesenchymal stem cells (hMSCs) were derived and donated by Dr. Xia [12].

### Generating integration-free hiPSCs and hiPSC culture

The human episomal vectors pEV SFFV-OS (OCT4-2a-SOX2), pEV SFFV-MK (MYC-2a-KLF4), and pEV SFFV-B (BCL-XL) were kindly donated by Dr. Xiao-Bing Zhang. PBMNCs were cultured for 5 days before nucleofection. To generate integration-free iPSCs, cells were nucleofected with 20 μg EV plasmid DNA (10 μg OS + 5 μg MK + 5 μg BCL-XL). PBMNCs (1.5 × 10^6^) were nucleofected by Amaxa Nucleofector® Program U-008 (Lonza) and then seeded into a 35-mm dish preseeded with feeder. The first colonies appeared at days 10–14 after coculture of PBMNCs with feeder. The number continued to increase. About 25 iPSC colonies were selected for further culture. The hiPSC medium was composed of knockout Dulbecco’s modified Eagle’s medium (DMEM; Invitrogen) supplemented with 15 % knockout serum replacement (KSR; Invitrogen), 5 % fetal bovine serum (FBS; Hyclone), 1 × nonessential amino acids (Invitrogen), 0.1 mM β-mercaptoethanol (Invitrogen), 1 mM L-glutamine (Invitrogen), and 8 ng/ml basic fibroblast growth factor (bFGF; Peprotech). iPSCs were passaged every 4–6 days by treatment with dispase (Invitrogen). After 10 passages, iPSCs were further characterized through karyotype analysis, pluripotency gene expression and cell differentiation ability (embryoid body (EB) and teratoma formation) examination.

### Karyotype analysis

One day after human iPSCs were subcultured, the cells were exposed to 0.25 μg/ml colcemid for 3.5 hours, digested, collected, and exposed to a hypotonic solution (0.4 % sodium citrate:0.4 % KCl = 1:1) for 16 minutes. The cells were fixed twice with methanol/acetic acid (3:1) for 1 hour in total. Then the cells were dropped onto cold, wet and clean glass slides. The slides were incubated for 4 hours at 70 °C. Then the slides were treated with 0.01 % trypsin at 37 °C for 10–12 seconds and washed with 0.9 % NaCl and then stained with Giemsa solution (Giemsa:phosphate buffer = 1.5 ml:40 ml, pH 7.4) at 37 °C for 2.5 minutes. The karyotype was determined by microscopic examination. More than twenty chromosomal spreads were counted per population.

### Immunocytochemical analysis

Colonies were fixed for 2 hours at room temperature with 4 % paraformaldehyde and then incubated at room temperature for 15 minutes with 1 % Triton X-100/phosphate-buffered saline (PBS). Cells were washed three times in PBS and blocked at 37 °C for over 3 hours with 4 % normal goat serum (Chemicon). Subsequently, cells were incubated at 4 °C overnight with primary antibody to Oct4 (1:500, Santa Cruz), SSEA4 (1:500, Life Technology), Nanog (1:500, Bethyl laboratories), TRA-1-60 (1:250, Life Technology), and TRA-1-81 (1:250, Life Technology). Cells were washed three times in PBS and incubated at 37 °C for 2 hours with goat anti-rabbit Alexa-Flour 594-conjugated (Life technologies) and goat anti-mouse Alexa-Fluor 488-conjugated (Life Technology) secondary antibodies (1:500 in 1 % normal goat serum in PBS). Unbound secondary antibodies were removed in three washes with PBS. Nuclei were identified by 1 μg/ml DAPI (Invitrogen) staining at room temperature for 5 minutes. Images were acquired using a confocal laser scanning microscope (LSM 510 META; Carl Zeiss).

### In vitro and in vivo differentiation

In vitro differentiation was performed by the EB formation method. The undifferentiated hiPSC colonies were manually dissected into smaller pieces using a fire-drawn glass needle. They were cultured in nonadherent petri dishes containing hiPSC medium without bFGF. EBs were characterized by the pieces of colonies taking on a round appearance, with smooth borders. Some irregularly shaped but smooth-bordered EBs may be several EBs clustered together.

In vivo differentiation was established by formation of teratomas and examined by hematoxylin and eosin staining. The undifferentiated hiPSC colonies from a confluent 60-mm dish were manual dissected into smaller pieces. Cells were suspended in 200 μl PBS containing 2 % KSR and were injected under the inguinal skin of SCID mice. After 7–8 weeks the teratomas were excised and fixed in 10 % neutral buffered formalin for 24 hours. Dehydration was performed with graded ethanol series followed by three consecutive steps of clarification in xylene and paraffin-embedment during tissue processing. Approximately 5 μm adjacent sections were made using a microtome. The slides were allowed to dry overnight at 42 °C. Deparaffinization was performed in two cycles using xylene. Decreasing alcohol series were used for rehydration, followed by a final rinse with deionized water for 5 minutes. Hematoxylin solution was added to the tissue for 1 minute. The sections were stained blue in NaOH until the nuclei stained bluish-purple and detected under microscopy. Sections were placed in deionized water for 3 minutes after being washed in distilled water. Dehydration was performed with graded ethanol series. Eosin solution was added to the tissue for 1 minute. Dehydration was performed with 100 % EtOH followed by clarification with xylene. The slides were mounted with resinous mounting medium and visualized under a microscope.

### Cell pellet formation and chondrocyte differentiation

The undifferentiated hiPSC colonies were manually dissected into smaller pieces and cultured for 10 days in nonadherent petri dishes containing hiPSC medium without bFGF for EB formation. Approximately 25 % of the initial media was replaced with an equal amount of the differentiation media (DMEM, 20 % FBS, 1 × non-essential amino acids, 0.1 mM β-mercaptoethanol, 1 mM L-glutamine) every 2 days. EBs were then seeded onto 10 cm gelatin-coated dishes. Within 10 days of cellular confluence, the cells were incubated with 0.25 % trypsin/EDTA at 37 °C for 5 minutes, and reseeded on new gelatin-coated dishes. At 90–100 % confluence of cells, which is often observed in 5 to 7 days, the cells were harvested with 0.25 % trypsin/EDTA. The cells were sorted by CD73 and CD105 double positivity. Then they (3 × 10^5^) were placed in a 15-ml polypropylene tube, centrifuged at 1200 rpm for 3 minutes at room temperature, and resuspended in chondrogenic differentiation medium (DMEM-HG supplemented with 10 % ITS (Invitrogen), 10^−7^ M dexamethasone, 1 mM ascorbate-2-phosphate (Invitrogen), 1 % sodium pyruvate (Invitrogen), and 10 ng/ml transforming growth factor-beta 1 (Peprotech)). The cells were recentrifuged and maintained in small pellet form for 21 days. The culture medium was replaced every 3 days. The hMSCs were also collected and cultured in this chondrogenic differentiation medium for 21 days.

### Flow cytometry analysis

Before cells were placed in a 15 ml polypropylene tube, the cell populations acquired a homogenous, fibroblast-like morphology. The iPSC–MSC-like cells were grown to attain confluence, harvested by 0.25 % trypsin/EDTA, washed with PBS, and resuspended in staining buffer consisting of 2 % FBS in PBS. Cell suspensions were mixed with PE mouse anti-human CD73, APC mouse anti-human CD105, APC mouse anti-human CD45 and PE mouse anti-human CD34. The isotype controls used were mouse IgG1 K isotype control APC and mouse IgG1 K isotype control PE, which were appropriate isotypes related to antibodies (eBioscience) in our experiments. Samples were run on a LSR II Flow Cytometer (BD Biosciences) instrument. For each analysis, a minimum of 10,000 cells were assayed. hMSCs were also used as a positive control and detected.

### Histological analysis of chondrogenic differentiation

Chondrogenic differentiation was assessed by alcian blue and toluidine blue staining of pellet sections. The aggregates were fixed in 10 % neutral-buffered formalin for 24 hours. Dehydration was performed with graded ethanol series followed by three consecutive steps of clarification in xylene and paraffin-embedment during tissue processing. Approximately 4-μm adjacent sections were made using a microtome. The slides were allowed to dry for 2 hours at a temperature of 60 °C. Deparaffinization was performed over three cycles using xylene. Decreasing alcohol series were used for rehydration, followed by a final rinse with deionized water for 5 minutes. The sections were then stained with 0.1 % alcian blue reagent or 1 % toluidine blue staining for 4–5 hours and were rinsed with distilled water. Dehydration was performed with graded ethanol series followed by three consecutive steps of clarification in xylene and the slides were mounted in Permount® (Fisher) and visualized under a microscope. Additional chondrogenic differentiation sections were further assessed by immunohistochemistry.

### Immunohistochemistry

Pellet sections for immunohistochemical staining were performed with a sequence of treatments as for the histological analysis. The sections were transfered onto glass slides suitable for immunohistochemistry examination. After deparaffinization and rehydration, we brought the slides to a boil in 1 mM EDTA pH 8.0 followed by 8 minutes at a sub-boiling temperature and then allowed the slides to cool at room temperature. The slides were rinsed with deionized water three times. Endogenous peroxidase activity was blocked by incubating sections in 3 % H_2_O_2_ solution in methanol at room temperature for 15 minutes. The slides were rinsed with deionized water and immersed with PBS for 5 minutes. Appropriately diluted primary antibody (50–100 μl) was applied to the sections on the slides and then they were incubated in a humidified chamber at room temperature for 1 hour. The following primary antibodies were applied: rabbit polyclonal antibodies against collagen II (1:50, Abcam) or mouse monoclonal antibodies to collagen X (1:50, Abcam). The slides were washed three times with PBS, 5 minutes each time. The samples were then incubated with the corresponding secondary antibodies (anti-rabbit and mouse; Roche) for 15 minutes at room temperature. The slides were further washed three times with PBS, 5 minutes each time, followed by DAB (Roche) detection under microscopy. The slides were washed three times with PBS, 2 minutes each time. The cell nuclei were counterstained by immersing the slides in hematoxylin for about 1–2 minutes. Dehydration was performed with graded ethanol series followed by three consecutive steps of clarification with xylene and the slides were finally mounted in Permount® (Fisher) and visualized under a microscope. Negative controls were processed following the same procedure omitting the primary antibodies to exclude the level of background staining.

### Sulfated glycosaminoglycan content

Sulfated glycosaminoglycan (sGAG) content of chondrogenic pellets was analyzed using the dimethylmethylene blue (DMMB; Sigma) spectrophotometric method. Cells or chondrogenic pellets were digested in 50 μl papain solution (10 U/mL in PBS with 0.1 M sodium acetate, 2.4 mM EDTA, 5 mM L-cysteine; Sigma) at 60 °C for 2 hours. sGAG content in sample digest (50 μl) was measured by mixing with DMMB dye solution (1 ml of 16 mg/l 1,9-dimethylmethylene blue, 40 mM glycine, 400 mM NaCl, pH 3.0) and measuring absorbance (525 nm). The concentration of sGAG in each sample was calculated against a standard curve of shark chondroitin sulfate (Sigma). DNA content was determined using the Qubit dsDNA HS assay kit and Qubit Fluorometer system (Invitrogen).

### In vivo chondrogenesis study

Six-week-old SCID mice were intraperitoneally anesthetized using avertin (1.25 %, Sigma). hiPSC–MSC-like cells, which were chondrogenically induced for 10 days in vitro, were implanted into the kidney capsule of the mice. The specimens were harvested 6 weeks after implantation. These specimens were fixed in 10 % neutral-buffered formalin, dehydrated, clarified, and embedded in paraffin. Sections were subjected to standard procedures with alcian blue staining, toluidine blue staining and collagen II and X immunochemical staining.

### Real-time polymerase chain reaction analysis

Total RNA was extracted by TRIzol reagent (Invitrogen). RNA was then converted into cDNA using the Reverse Transcriptase System (Promega). cDNA samples were subjected to real-time polymerase chain reaction (PCR) with KAPA SYBR FAST qPCR kit Master Mix (2×) using a real-time PCR System. The primer sequences were: *hAGGRECAN*-F:TCGAGGACAGCGAGGCC, *hAGGRECAN*-R: TCGAGGGTGTAGCGTGTAGAGA; *hβ-ACTIN*-F: TTTGAATGATGAGCCTTCGTCCCC, *hβ-ACTIN*-R: GGTCTCAAGTCAGTGTACAGGTAAGC; *hCOL2*-F:TGGACGATCAGGCGAAACC, *hCOL2*-R:GCTGCGGATGCTCTCAATCT; *hSOX9*-F:AGCGAACGCACATCAAGAC, *hSOX9*-R:CTGTAGGCGATCTGTTGGGG; *hCOL10*-F: ATGCTGCCACAAATACCCTTT, *hCOL10*-R: GGTAGTGGGCCTTTTATGCCT. The 20 μl PCR systems contained 1 μl cDNA template, SYBR Green qPCR Master Mix, 0.25 mM forward primer and reverse primer. The reactions were incubated in a 96-well plate at 95 °C for 10 minutes, followed by 40 cycles of 95 °C for 15 seconds and 60 °C for 1 minute. All reactions were repeated three times. β-actin was used as an internal control.

### Statistical analysis

All quantitative data were collected independently at least three times and yielded reproducible results. Data are presented as the mean ± standard error of the mean (SEM). Statistical analysis was performed using one-way analysis of variance. Differences were considered statistically significant at *P* < 0.05.

## Results

### Generation of hiPSCs from peripheral blood

**Fig. 1:**
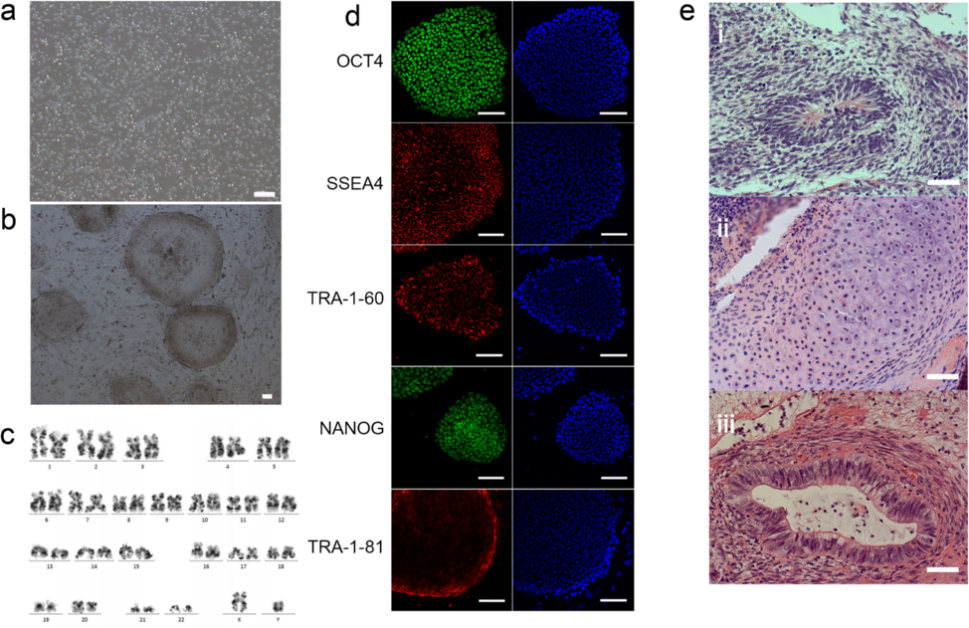
Generation of hiPSCs from peripheral blood. **a** Phase-contrast image of the PBCs. Scale bar = 100 μm. **b** Phase-contrast image of a hiPSC line. Scale bar = 100 μm. **c** Karyotype (46,XY). **d** Fluorescent immunostaining of pluripotent markers OCT4, SSEA4, TRA-1-60, NANOG, and TRA-1-81. Scale bars = 100 μm. **e** Teratoma formation. Teratomas were harvested over 2 months after subcutaneous injection of the iPSCs into SCID mice. (i) Neuroepithelial cells, (ii) cartilage, and (iii) respiratory epithelium were detected by hematoxylin and eosin staining. Scale bar = 50 μm

We generated hiPSCs by episomal reprogramming, which was virus-free, nonintegrated and clinically relevant. Human PBCs (Fig. 1a) were transfected with modified episomal vectors. About 13–14 days after transfection, the human embryonic stem cell-like colonies (iPSC clones) became visible. On day 24, more than 40 iPSC clones were observed. The iPSC clones were then selected and harvested for expansion (Fig. 1b). Most of the iPSCs were karyotypically normal (46,xy) (Fig. 1c). The iPSC lines used in this research were positive for the pluripotent markers OCT4, NANOG, SSEA4, TRA-1-60 and TRA-1-81(Fig. 1d). Teratomas were harvested over 2 months after the subcutaneous injection of iPSCs into SCID mice. Histological examination showed that the teratoma contained various tissues of the three germ layers (Fig. 1e).

### Mesenchymal differentiation of hiPSCs

**Fig. 2:**
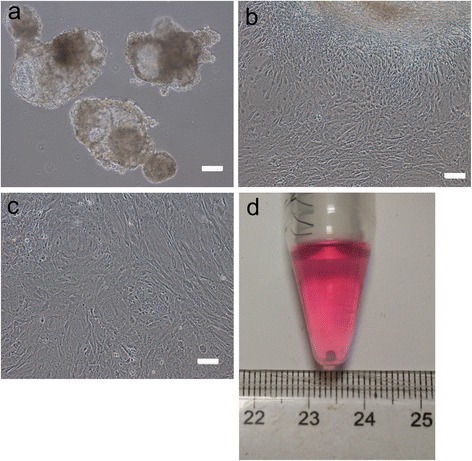
Generation of hiPSC–MSC-chondrogenic pellets via EB formation, monolayer cell culture and three-dimensional pellet culture. **a** EB formation. Scale bar = 100 μm. **b** Cell outgrowth from EBs. Scale bar = 100 μm. **c** Monolayer cell culture. Scale bar = 100 μm. **d** Three-dimensional pellet culture

In order to differentiate the hiPSCs into the mesenchymal lineage, we changed the culture medium of hiPSCs into the differentiation medium, free of bFGF. A multi-step culture method combining EB formation, monolayer culture and expansion were used as follows. First, the iPSCs were spontaneously differentiated via EB formation (Fig. 2a), followed by cell outgrowth from EBs on gelatin-coated dishes. Typical iPSC morphology was gradually lost and a spindle-shaped morphology at the border of the colonies was formed (Fig. 2b). After 20 days of differentiation, the cells were passaged with trypsin/EDTA and the morphological change of the cells to a fibroblastic shape was observed after passage. The cells were then cultured and expanded in monolayer after subculture (Fig. 2c). The monolayer step was performed to exclude residual undifferentiated cells present in the EBs as well as to expand cells committed to the mesenchymal lineage. The cells were seeded and cultivated in monolayers for 5–7 days until cells reached about 90 % confluence. Most cells were hiPSC–MSC-like cells. Finally a thre-dimensional pellet culture was utilized for chondrocyte differentiation (Fig. 2d) [13].

### Characterization of hiPSC–MSC-like cells

**Fig. 3:**
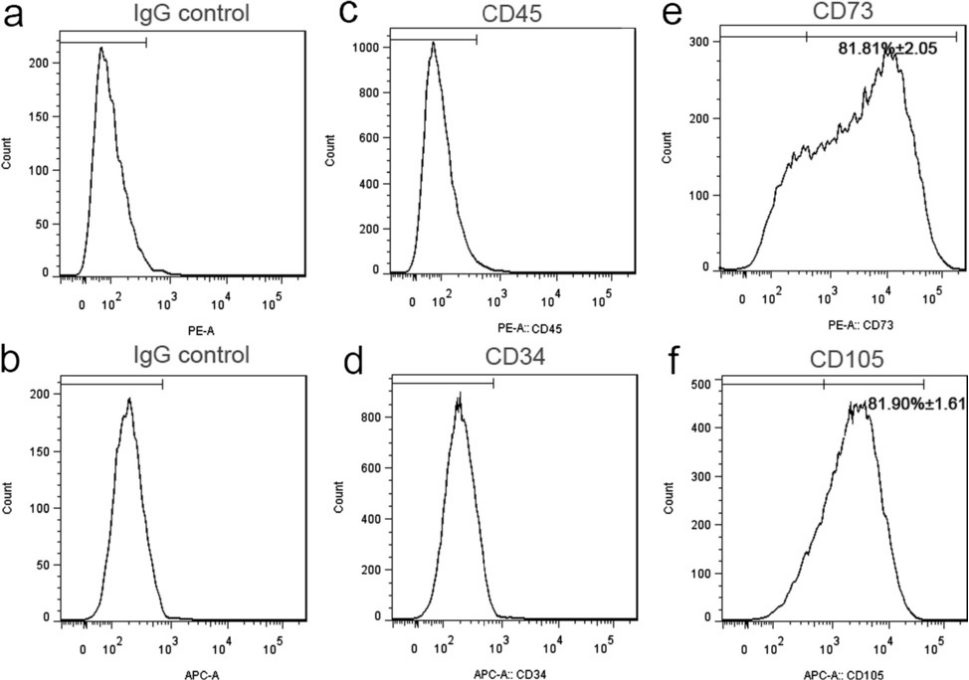
Flow cytometric analysis of the MSC markers (CD73, CD105) and hematopoietic markers (CD34, CD45) in monolayer-cultured hiPSC–MSC-like cells. **a** Immunological control for CD45, CD73. **b** Immunological control for CD34, CD105. **c**, **d** The cell surface markers CD34 and CD45 were not expressed. **e** The proportion of CD73-expressing cells was 81.81 ± 2.05 %. **f** The proportion of CD105-expressing cells was 81.90 ± 1.61 %. Values represent means ± SEM; n = 3. *CD* cluster of differentiation, *Ig* immunoglobulin

Before proceeding to pellet culture, the expression of cell surface markers on hiPSC–MSC-like cells were analyzed. Flow cytometric analysis demonstrated that the majority of hiPSC–MSC-like cells expressed CD73 (81.81 ± 2.05 %) and CD105 (endoglin) (81.90 ± 1.61 %), which are known to be positive for human mesenchymal markers. CD34 and CD45, hematopoietic markers that are not expressed in MSCs, were not detected in monolayer-cultured hiPSC–MSC-like cells (Fig. 3). Expression of CD73, CD105 and the lack of CD34 and CD45 suggested that monolayer-cultured hiPSC–MSC-like cells may commit to differentiating into mainly mesenchymal lineage cells. As a comparison, we also detected the expression of CD73, CD105, CD34, CD45, TRA-1-60 and SSEA4 of hiPSCs (Additional file 1: Figure S1) and the expression of CD73, CD105, CD34 and CD45 of hMSCs (Additional file 2: Figure S2a). Almost all the hiPSCs expressed TRA-1-60 (94.8 ± 0.57 %) and SSEA4 (99.0 ± 0.12 %), and all the hMSCs expressed CD73 (99.7 ± 0.63 %) and CD105 (98.7 ± 1.21 %). In order to avoid off-target effects, we sorted pure MSCs (CD73 and CD105 double positive cells) (Additional file 3: Figure S3) for chondrogenic differentiation.

### Morphological and histological characterization of hiPSC–MSC chondrogenic pellets

**Fig. 4:**
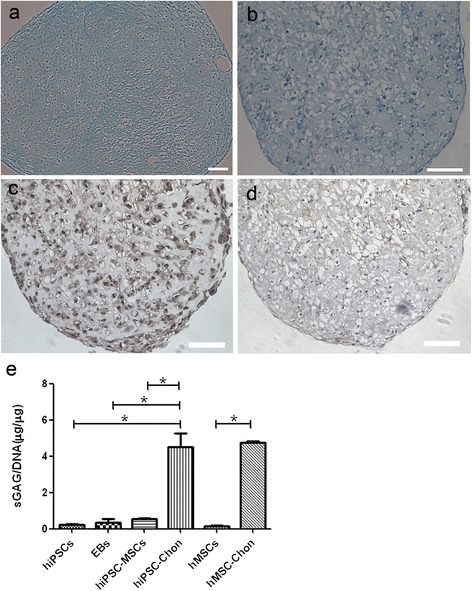
Characterization of hiPSC–MSC chondrogenic pellets. **a** Alcian blue staining and **b** toluidine blue staining of glycosaminoglycans and proteoglycans revealed the chondrocyte-type appearance of the hiPSC–MSC chondrogenic pellets. Scale bars = 100 μm. **c**, **d** Immunohistochemistry for type II and type X collagen. Scale bars = 100 μm. **e** Biochemical characterization of hiPSC–MSC chondrogenic pellets (hiPSC-Chon) versus hiPSCs, EBs, hiPSC–MSC-like cells (hiPSC–MSCs) and hMSC chondrogenic pellets (hMSC-Chon) versus hMSCs. sGAG per DNA. Bars represent means ± SEM; n = 3; *P < 0.05. *EB* embryoid body, *hiPSC* human induced pluripotent stem cell, *hMSC* human mesenchymal stem cell, *sGAG* sulfated glycosaminoglycan

After monolayer-cultured hiPSC–MSC-like cells were dissociated into single cells, CD73^+^CD105^+^ cells were sorted and cultured in 15-ml polypropylene tubes in pellets for 21 days. Under these conditions, chondrogenic cells are known to self-assemble in vitro into cartilaginous tissue containing characteristic components of extracellular matrix. In our experiments, dense cartilage-like aggregates were observed. These aggregates were up to 2–3 mm long and 3 mm thick (Fig. 2d). Paraffin sections of the aggregates were stained with alcian blue and toluidine blue, which detected glycosaminoglycans and proteoglycans typical of the cartilage extracellular matrix (glycoprotein-rich matrix). The cell morphology and positive staining for alcian blue (Fig. 4a) and toluidine blue (Fig. 4b) indicated the chondrogenic differentiation of hiPSC–MSC pellets. The extracellular matrix of the hiPSC–MSC chondrogenic pellets were further examined for the staining of type II and X collagen by immunohistochemistry. Immunostaining positive for collagen II (Fig. 4c) and collagen X (Fig. 4d) showed the hiPSC–MSC chondrogenic pellets had developed a chondrocyte-like phenotype. The negative control (Additional file 4: Figure S4a) was shown to exclude the level of background staining and better prove the positive immunostaining.

After 21 days of chondrogenic induction, hMSCs also formed dense cartilage-like aggregates (hMSC chondrogenic pellets), which were 2–3 mm thick (Additional file 2: Figure S2b) similar to the hiPSC–MSC chondrogenic pellets. Paraffin sections of the aggregates were positively stained with alcian blue and toluidine blue (Additional file 2: Figure S2c,d). Compared to the negative control (Additional file 4: Figure S4b), the immunochemistry revealed that the pellets were surrounded by collagen II- and X-positive extracellular matrix (Additional file 2: Figure S2e,f).

### Biochemical analysis of hiPSC–MSC chondrogenic pellets

sGAG analysis was also performed after 21 days of chondrogenic differentiation (Fig. 4e). hiPSC–MSC chondrogenic pellets were compared with hiPSC–MSC-like cells and hMSC chondrogenic pellets, as well as the undifferentiated hiPSCs and EBs. sGAG content was significantly increased in hiPSC–MSC chondrogenic pellets compared to hiPSC–MSC-like cells, as well as undifferentiated hiPSCs and EBs (*P* < 0.05). As a comparison, the sGAG content of hMSC chondrogenic pellets was also significantly higher than hMSCs (*P* < 0.05), but was not significantly different to hiPSC–MSC chondrogenic pellets.

### In vivo chondrogenesis study

To determine the chondrogenic ability of hiPSC–MSC-like cells to differentiate in vivo, we implanted hiPSC–MSC-like cells into the kidney capsule of the SCID mice. These in vitro differentiated cells continued to proliferate and differentiate in vivo. The pellets were harvested 6 weeks after transplantation. The morphology and positive staining for alcian blue and toluidine blue, and the staining of type II and X collagen-positive extracellular matrix (Additional file 5: Figure S5) of the pellet, indicated the chondrogenic differentiation of hiPSC–MSC-like cells in vivo.

### Gene expression of chondrogenic differentiation markers in hiPSC–MSC chondrogenic pellets

**Fig. 5:**
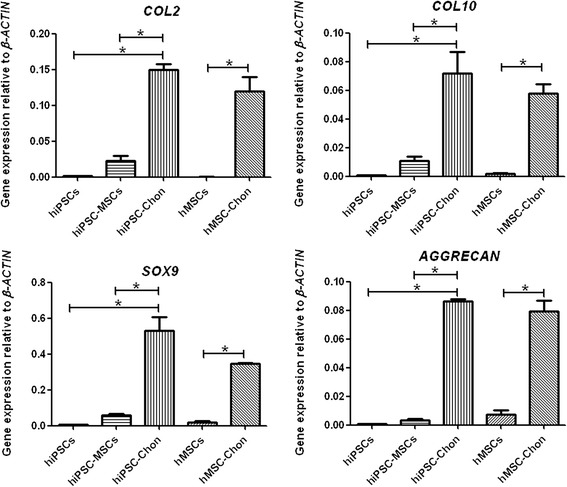
Gene expressions of chondrogenic markers in hiPSC–MSC chondrogenic pellets. The expressions of *COL2, COL10, SOX9* and *AGGRECAN* of hiPSC–MSC chondrogenic pellets (hiPSC-Chon) versus hiPSCs and hiPSC–MSC-like cells (hiPSC–MSCs), compared with hMSC pellets (hMSC-Chon) versus hMSCs. The expression levels of *COL2, COL10, SOX9* and *AGGRECAN* were analyzed by real-time quantitative PCR relative to *β-ACTIN*. Bars represent means ± SEM; n = 3; *P < 0.05. *hiPSC* human induced pluripotent stem cell, *hMSC* human mesenchymal stem cell

The phenotype of chondrogenic pellets was further characterized by assessing the relative expression of discrete genes representative of the chondro-progenitor lineage (*SOX9* and *COL2*) and fully differentiated chondrocytes (*AGGRECAN* and *COL10*) (Fig. 5). Transcript expressions of *AGGRECAN* and *COL10*, the definitive markers of differentiated chondrocytes, were more markedly increased in the hiPSC–MSC chondrogenic pellets than in hiPSCs and hiPSC–MSC-like cells (*P* < 0.05), which further suggested a more advanced chondrogenic differentiation. As a comparison, the markers for differentiated chondrocytes in hMSC chondrogenic pellets were significantly higher than in hMSCs (*P* < 0.05). These findings comprehensively confirmed a successful approach to exploiting chondrogenic differentiation via hiPSCs.

## Discussion

Limited seed cell numbers remains a significant challenge for cartilage tissue regeneration. Scientists have tried various cells sources for cartilage tissue repair. Previous studies showed that PBCs may be a good candidate for stem cell harvest, but inadequate information on how to change or transform them into chondrocytes still remains. In this study, we successfully transformed PBCs into chondrocytes through an integration-free method. Our findings suggested that, with appropriate differentiation and purification protocols, human PBCs could be reprogrammed into iPSCs and further differentiated into chondrogenic lineage in vitro. Similar to fibroblasts, four steps were taken after PBCs were reprogrammed into iPSCs: (1) EB formation; (2) cell outgrowth from EBs to increase cell number; (3) monolayer culture to remove undifferentiated cells and choose cells that can follow MSC growth conditions; and (4) three-dimensional pellet culture. When MSC-like cells were generated, their surface markers were analyzed. The results showed positivity for CD73 and CD105, and negativity for CD34 and CD45, similar to murine MSC induction from iPSCs [14]. In the consequent pellet culture medium, transforming growth factor-beta1 and dexamethasone were supplemented. These factors have been classically demonstrated to hold strong influence over the chondrogenic potential ability [15].

MSCs are considered to be the current leading regenerative therapeutic cells for musculoskeletal diseases, and various studies have been conducted. However, there are still limiting factors such as heterogeneity, insufficient cell numbers, and limited proliferative potential. Our study showed that the generation of iPSCs from human PBCs may provide an alternative source of MSCs for cartilage repair. Blood-derived iPSCs, which have been proven to differentiate into MSCs, hepatocytes and even cardiomyocytes, showed potential regenerative abilities. Furthermore, PBCs may serve as an ideal source for cellular reprogramming and cartilage tissue repair. As peripheral blood is abundant and readily available, a good amount of cells could be harvested from a given blood volume. Additionally, PBCs do not require extensive cell culture before they undergo reprogramming [16]. Nowadays, peripheral blood application is common and is widely used in the area of medical diagnostics and treatment. Furthermore, large numbers of frozen blood samples are stored in biorepositories ready for use when needed. Nucleated blood cells are essential cells in the blood for reprogramming, including T lymphocytes, B lymphocytes and other progenitor cells, while red blood cells and platelets are depleted using lytic buffer treatment often following multiple centrifugations, after which a concentration of mononuclear cells can be obtained.

So far, viral and nonviral vector approaches have been adopted for the generation of iPSCs. In early studies, viral vectors were commonly used to convert blood CD34^+^ cells into iPSCs due to their high-level reprogramming efficiencies [9]. However, several safety concerns, such as insertional mutagenesis, tumor formation and differentiation process interruption, and so forth, have been previously proposed [17, 18]. Therefore, integration-free iPSCs might have the potential to eliminate the disadvantages associated with integrating virus-associated genotoxicity and may be a promising direction for clinical applications. Although many techniques could be used to generate iPSCs from fibroblasts, there have been only two approaches so far reported that could be employed and adopted in generating blood-derived integration-free iPSCs: the sendai virus vector and episomal vector (EV) approaches. In this study, we utilized EV, adopting the method of Su et al. [5]. EV seems to be a cost-effective approach that requires no need for viral vector packaging, and can be achieved by a single infection.

The disadvantage associated with nonviral generation is mostly reported to be the reprogramming efficiency. Okita et al. [19] found that efficiency from mononuclear cells could reach up to 0.1 % maximum, which suggested that only a small volume of peripheral blood was sufficient for the induction into iPSCs. They also found frozen cells could further be utilized for the induction [19]. In this study, the reprogramming efficiency in generating integration-free iPSCs from blood cells was also satisfactory and conserved. More than 40 iPSCs were easily generated from a 2 ml volume of peripheral blood, which was adequate enough for this experiment. This is consistent with the findings of Su et al. who demonstrated that about 20 to 30 iPSCs could be generated from a 1 ml peripheral blood sample [5]. This is evidence that the nonviral method is promising for future clinical applications.

Differentiation of iPSCs toward the chondrogenic lineage is usually a complex process. In view of this, classic chondrogenic medium was utilized for the induction of chondrogenesis from hiPSCs. In our study, the specific matrix composition and the expression of sequential markers of chondrogenic differentiation indicate successful induction. Transcript expressions of *AGGRECAN* and *COL10*, the definitive markers of fully differentiated chondrocytes, were also markedly increased in the hiPSC–MSC chondrogenic pellets.

Our study found both chondrogenic gene expression levels and sGAG production between hiPSC–MSC pellets and hMSC pellets to be similar. Kang et al. also found that hiPSC–MSCs have advanced proliferation capability and adequate osteogenic and chondrogenic properties compared to MSCs [20]. In vivo assay also indicated chondrogenic differentiation of hiPSC–MSC-like cells. Our findings suggested blood cell-derived hiPSC–MSCs possess a potential chondrogenic ability similar to bone marrow-derived hMSCs.

In our study, collagen type X expression in both hiPSC–MSC chondrogenic pellets and hMSC chondrogenic pellets generated in vitro was weak, while aggrecan and collagen II were robustly expressed. The low levels of collagen type X staining in vitro were consistent with the hyaline cartilage phenotype. Longer culture in vivo resulted in substantial type X collagen staining, which is indicative of endochondral progression. Pelttari et al. have reported that MSC pellets with 4–7 week induction were also positive for type X collagen [21], while chondrocytes from permanent articular cartilage remain negative for collagen type X [22]. As hiPSC–MSCs may possess similar chondrogenic ability to hMSCs, it would also be expected that they would express chondrogenic markers similarly.

## Conclusions

In conclusion, this study proves that PBCs can be utilized as candidates for regenerated chondrocytes. This could reflect a future direction to generate seed cells for cartilage repair in a patient-specific approach with cost-effective applications in regenerative medicine.

## Additional files

Additional file 1: Figure S1. Flow cytometric analysis of the pluripotent markers (TRA-1-60, SSEA4), hematopoietic markers (CD34, CD45) and MSC markers (CD73, CD105) in hiPSCs. The proportion of TRA-1-60-expressing cells was 94.8 ± 0.57 %; The proportion of SSEA4-expressing cells was 99.0 ± 0.12 %. Values represent means ± SEM; n = 3. (TIF 44 kb) Additional file 2: Figure S2. Characterization of hMSCs and hMSC-chondrogenic pellets. (a) Flow cytometric analysis of the hematopoietic markers (CD34, CD45) and MSC markers (CD73, CD105) in hMSCs. The proportion of CD73-expressing cells was 99.7 ± 0.63 %; The proportion of CD105-expressing cells was 98.7 ± 1.21 %. Values represent means ± SEM; n = 3. (b) Three-dimensional pellet culture of the hMSC-chondrogenic pellet. (c) Alcian blue staining and (d) toluidine blue staining of glycosaminoglycans and proteoglycans revealed the chondrocyte-type appearance in the hMSC-chondrogenic pellets. Scale bar = 100 μm. (e,f) Immunohistochemistry for type II and type X collagen. Scale bar = 100 μm. (TIF 8130 kb) Additional file 3: Figure S3. Flow cytometric analysis of the MSC markers (CD73, CD105) on a single scatter plot. CD73 and CD105 double-positive cells (about 70.2 %) were sorted for chondrogenic differentiation. (TIF 731 kb) Additional file 4: Figure S4. Negative controls of immunohistochemistry for type II and type X collagen. The slides were performed omitting the primary antibodies. As the secondary antibody is commonly used for anti-rabbit and mouse, so the negative controls for collagen type II and type X are the same. (a,b) Negative control for immunohistochemistry of hiPSC–MSC chondrogenic pellets and hMSC chondrogenic pellets. Scale bar = 100 μm. (TIF 8652 kb) Additional file 5: Figure S5. Characterization of hiPSC–MSC chondrogenic pellets cultured in vivo. (a) Alcian blue staining and (b) toluidine blue staining of glycosaminoglycans and proteoglycans revealed the chondrocyte-type appearance in the pellets. Scale bar = 100 μm. (c,d) Immunohistochemistry for type II and type X collagen. Scale bar = 100 μm. (TIF 7452 kb)

## Abbreviations

bFGF: Basic fibroblast growth factor
DMEM: Dulbecco’s modified Eagle’s medium
DMMB: Dimethylmethylene blue
EB: Embryoid body
EV: Episomal vector
FBS: Fetal bovine serum
hiPSC: Human induced pluripotent stem cell
hMSC: Human mesenchymal stem cell
IRB: Institutional Review Board
KSR: Knockout serum replacement
MSC: Mesenchymal stem cell
PBC: Peripheral blood cell
PBMNC: Peripheral blood mononuclear cell
PBS: Phosphate-buffered saline
PCR: Polymerase chain reaction
SEM: Standard error of the mean
sGAG: Sulfated glycosaminoglycan
