# Supplementary figures and images for: Reprogramming of blood cells into induced pluripotent stem cells as a new cell source for cartilage repair
Source: Stem Cell Res Ther. 2016 Feb 17;7:31. doi: 10.1186/s13287-016-0290-7 (PMC4756426; doi:10.1186/s13287-016-0290-7)

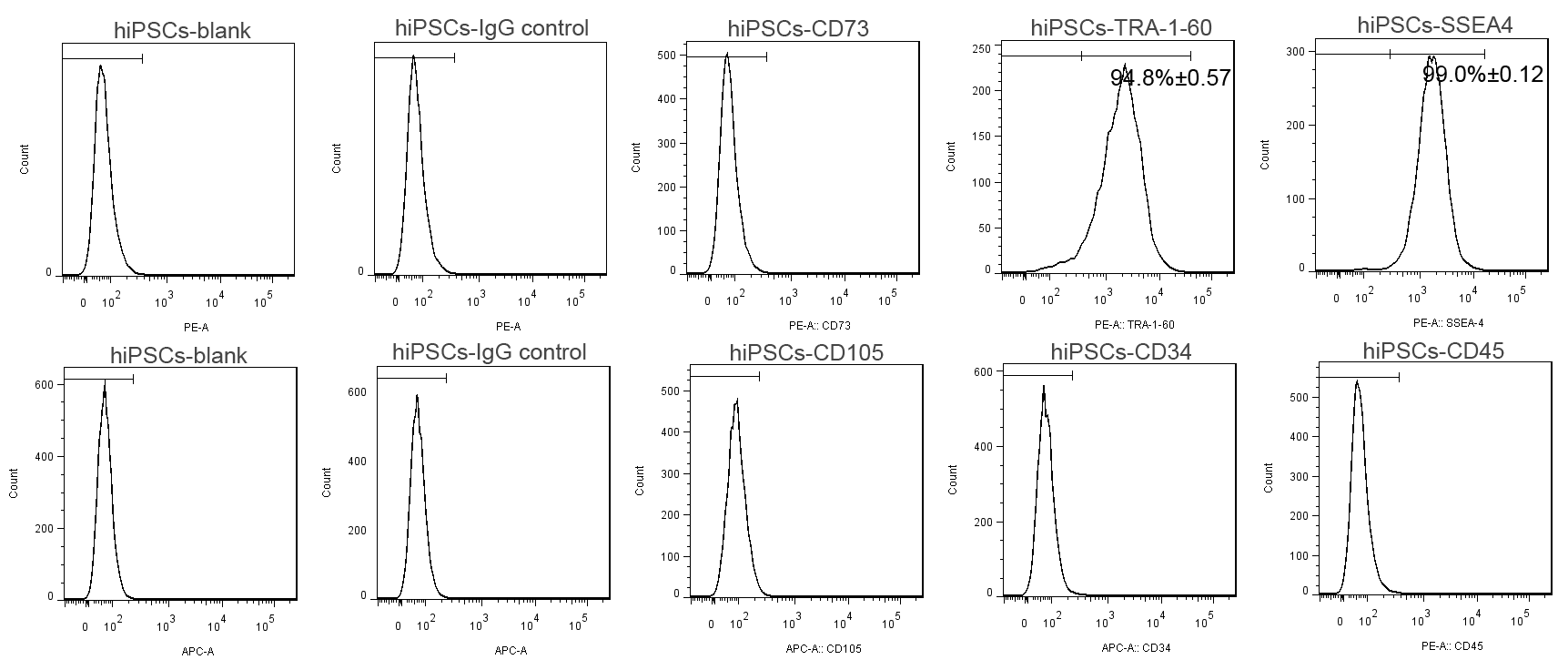

Supplement: Additional file 1: Figure S1. — Flow cytometric analysis of the pluripotent markers (TRA-1-60, SSEA4), hematopoietic markers (CD34, CD45) and MSC markers (CD73, CD105) in hiPSCs. The proportion of TRA-1-60-expressing cells was 94.8 ± 0.57 %; The proportion of SSEA4-expressing cells was 99.0 ± 0.12 %. Values represent means ± SEM; n = 3. (TIF 44 kb) [file 13287_2016_290_MOESM1_ESM.tif]

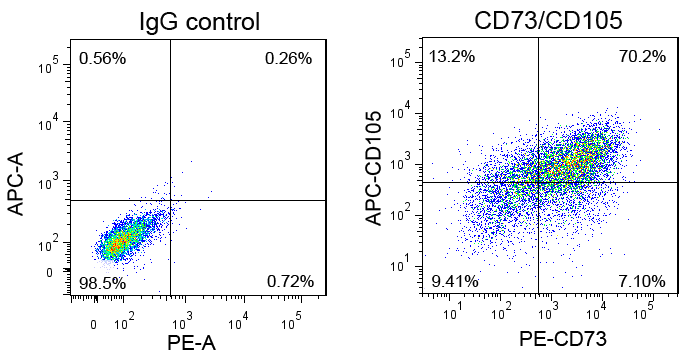

Supplement: Additional file 3: Figure S3. — Flow cytometric analysis of the MSC markers (CD73, CD105) on a single scatter plot. CD73 and CD105 double-positive cells (about 70.2 %) were sorted for chondrogenic differentiation. (TIF 731 kb) [file 13287_2016_290_MOESM3_ESM.tif]
